# Phrenic nerve stimulation to prevent diaphragmatic dysfunction and ventilator-induced lung injury

**DOI:** 10.1186/s40635-023-00577-5

**Published:** 2023-12-18

**Authors:** Alessandro Panelli, Michael A. Verfuß, Martin Dres, Laurent Brochard, Stefan J. Schaller

**Affiliations:** 1 Charité - Universitätsmedizin Berlin, Department of Anesthesiology and Intensive Care Medicine (CCM/CVK), Berlin, Germany; 2 Sorbonne Université, INSERM UMRS 1158, Neurophysiologie Respiratoire Expérimentale et Clinique, Paris, France; 3 Service de Médecine Intensive et Réanimation, Département R3S, APHP, Sorbonne Université, Hôpital Pitie Salpêtrière, Paris, France; 4 Unity Health Toronto, Keenan Research Centre for Biomedical Science, Li Ka Shing Knowledge Institute, Toronto, ON Canada; 5 Interdepartmental Division of Critical Care, University of Toronto, Toronto, Canada; 6 Technical University of Munich, School of Medicine and Health, Klinikum Rechts der Isar, Department of Anesthesiology and Intensive Care Medicine, Munich, Germany

## Abstract

Side effects of mechanical ventilation, such as ventilator-induced diaphragmatic dysfunction (VIDD) and ventilator-induced lung injury (VILI), occur frequently in critically ill patients. Phrenic nerve stimulation (PNS) has been a valuable tool for diagnosing VIDD by assessing respiratory muscle strength in response to magnetic PNS. The detection of pathophysiologically reduced respiratory muscle strength is correlated with weaning failure, longer mechanical ventilation time, and mortality. Non-invasive electromagnetic PNS designed for diagnostic use is a reference technique that allows clinicians to measure transdiaphragm pressure as a surrogate parameter for diaphragm strength and functionality. This helps to identify diaphragm-related issues that may impact weaning readiness and respiratory support requirements, although lack of lung volume measurement poses a challenge to interpretation. In recent years, therapeutic PNS has been demonstrated as feasible and safe in lung-healthy and critically ill patients. Effects on critically ill patients’ VIDD or diaphragm atrophy outcomes are the subject of ongoing research. The currently investigated application forms are diverse and vary from invasive to non-invasive and from electrical to (electro)magnetic PNS, with most data available for electrical stimulation. Increased inspiratory muscle strength and improved diaphragm activity (e.g., excursion, thickening fraction, and thickness) indicate the potential of the technique for beneficial effects on clinical outcomes as it has been successfully used in spinal cord injured patients. Concerning the potential for electrophrenic respiration, the data obtained with non-invasive electromagnetic PNS suggest that the induced diaphragmatic contractions result in airway pressure swings and tidal volumes remaining within the thresholds of lung-protective mechanical ventilation. PNS holds significant promise as a therapeutic intervention in the critical care setting, with potential applications for ameliorating VIDD and the ability for diaphragm training in a safe lung-protective spectrum, thereby possibly reducing the risk of VILI indirectly. Outcomes of such diaphragm training have not been sufficiently explored to date but offer the perspective for enhanced patient care and reducing weaning failure. Future research might focus on using PNS in combination with invasive and non-invasive assisted ventilation with automatic synchronisation and the modulation of PNS with spontaneous breathing efforts. Explorative approaches may investigate the feasibility of long-term electrophrenic ventilation as an alternative to positive pressure-based ventilation.

## Take-home message

Diaphragm stimulation in critically ill patients using phrenic nerve stimulation may reduce diaphragm atrophy and has been indicated to improve functionality, i.e. respiratory muscle strength. In the future, ventilator-induced adverse events might be reduced by automatic, spontaneous breathing synchronised phrenic nerve stimulation in combination with invasive or non-invasive ventilatory support forms.

## Background

Mechanical ventilation (MV) is a life-saving intervention for critically ill patients; however, it comes with a notable burden. This encompasses intensive care unit acquired weakness (ICUAW) [1] and ventilator-induced lung injury (VILI) [2].

Approximately 40–80% of patients admitted to the intensive care unit (ICU) are reported to experience at least one form of neuromuscular dysfunction [3, 4]. There is a wide range of neuromuscular dysfunction: critical illness polyneuropathy, critical illness myopathy, the combination critical illness polyneuromyopathy, and ventilator-induced diaphragm dysfunction (VIDD) [1]. VIDD is characterised by a reduction in diaphragm force-generating capacity [5], that may either coexist with ICUAW or manifest independently from it [6, 7]. VIDD often coincides with diaphragm atrophy developed under the exposure of mechanical ventilation in the ICU, together termed critical illness-associated diaphragm weakness [6].

VILI, on the other hand, represents the interplay of physical forces (inducing mechanical stress) and biological forces (inducing catabolic stress), which is associated with MV and injures the lungs [2].

Diaphragm weakness and VILI correlate with unfavourable outcomes [8]. So far, the only suggested therapy concept is lung-protective ventilation [9]. A potential future perspective to counteract and mitigate diaphragm weakness would be to promote diaphragm contraction, providing that lung distending pressures are safe. Prior studies have demonstrated that peripheral muscle stimulation prevents muscle atrophy in critically ill patients and seems to be able to preserve or increase muscle strength [10]. In contrast, there is still a lack of solid evidence regarding comparable morphological and functional evidence of diaphragm stimulation. Recently, non-invasive techniques and strategies to activate the diaphragm during MV by phrenic nerve stimulation (PNS) have been tested [11].

### Review scope and aims

This review aims to analyse the current evidence of PNS for preventing diaphragm weakness and explore the potential protection against VILI in critically ill patients.

### Definitions

Ventilator-induced diaphragm dysfunction: reduction in diaphragmatic force-generating capacity specifically related to the use of MV accompanied by diaphragm muscle inactivity and unloading [5].

Diaphragm atrophy: skeletal muscle wasting that occurs rapidly during critical illness or diaphragm inactivity with decreased protein synthesis and increased proteolysis [12–14].

Critical illness-associated diaphragm weakness refers to the insufficiency of the primary respiratory muscle due to its multiple causes. Therefore, this definition includes all forms of critical illness-associated diaphragmatic injury that lead to clinically measurable dysfunction or morphological alterations of the diaphragm [6].

Ventilator-induced lung injury (VILI): The concept of ventilator-induced lung injury (VILI) encompasses the various deleterious effects of MV on the lungs, in particular, *barotrauma* (lung distending pressure), *volutrauma* (lung stretching), *atelectrauma* (repetitive re-inflating) and *biotrauma* (systemic release of intracellular mediators) [2].

### Epidemiology and impact on clinical outcomes

Since VILI as a concept cannot be diagnosed in patients, epidemiological data are not available [15]. Undoubtedly, however, the outcome effect includes mortality from VILI [16].

Critical illness-associated diaphragm weakness in the form of contractile dysfunction represents a highly prevalent condition in mechanically ventilated patients (60–80%) [3, 4, 17]. Diaphragm weakness can occur independently from MV and can be related to sepsis, denutrition and medications [6]. It leads to weaning failure in over 50% of patients, extended stays in the ICU [18], and increased ICU mortality rates [19].

### Pathophysiology of ventilator-induced lung injury in the critically ill

Low lung-volume and high tidal-volume ventilation are suspected to induce different pathological processes. As a possible adverse effect, low-volume ventilation caused by insufficient PEEP may prompt atelectrauma by repeatedly reopening closed airways and re-inflating collapsed lung sections, causing epithelial damage and oedema. Conversely, high-volume ventilation increases the risk of barotrauma and volutrauma, accompanied by high pulmonary forces leading to lung stress and dynamic strain, consequently resulting in regional alveolar overdistention [2, 20]. Furthermore, respiratory system elastance and, hence, driving pressure interfere with disease severity and prognosis [21]. Increased alveolar-capillary permeability and oedema induced by the ventilator may result in biotrauma, involving the transfer of mediators, bacteria, and lipopolysaccharides across the air–blood barrier. Potential systemic inflammation and multi-organ failure due to the shift of pathogens can be a consequence [2], with sepsis being the leading cause of death in acute respiratory distress syndrome (ARDS) [22, 23]. This interplay of atelectrauma, barotrauma, volutrauma, and biotrauma forms a multifactorial injury leading to VILI.

### Pathophysiology of diaphragmatic changes in the critically ill

A rapid onset characterises diaphragm weakness in the critically ill. Structural damage incurred during MV follows a time-dependent trajectory: the duration of MV directly correlates with fibre injury and proteolysis [13], with a loss of over 50% of the cross-sectional area of diaphragmatic muscle fibres after only 18–69 h of MV [12]. Unloading the diaphragm results in contractile dysfunction with a significant reduction in force production [24]. Concerning the time to development of VIDD, no data from humans are available, but half of the patients will not use their diaphragm after intubation in the ICU, and some will take more than five days before they use their diaphragm again [25]. Animal models suggest an onset of diaphragm dysfunction after 12 h of MV [26, 27].

Diaphragm contractile activity contributes to the rate and direction of diaphragm thickness change. Low inspiratory effort leads to diaphragm atrophy, while excessive inspiratory effort due to a low level of ventilatory support is associated with increased diaphragm thickness, reflecting injury to the muscle [28]. These critical illness-associated deviations of diaphragmatic contractile activity caused thickness changes, independently from the direction of change, eventually leading to poor outcomes (i.e. prolonged MV duration, prolonged ICU and hospital stay, higher complications of acute respiratory failure with higher reintubation rates). Intermediate thickness and thickening fraction changes appear to be associated with better prognosis [14]. Thus, the diaphragm could be maintained in a healthy state by adequate diaphragm activity or training. Since the baseline state of patients has usually not been assessed beforehand, the status of the diaphragm at ICU admission is used currently, although this might be biased already, as previously shown [29].

**Fig. 1:**
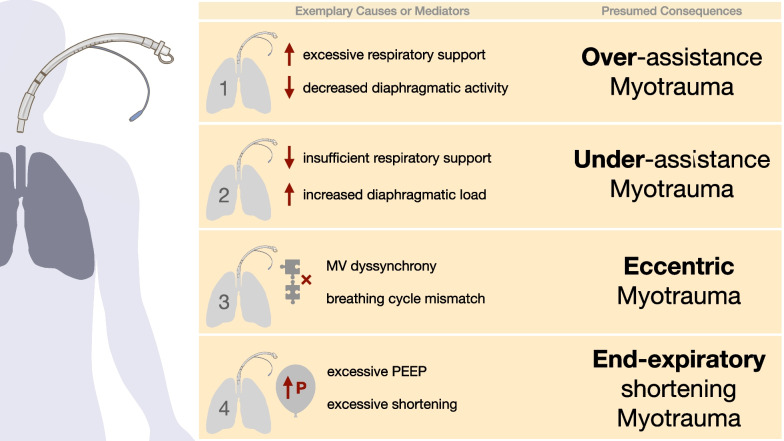
Possible causal factors (on the left) of the four types of ventilation-induced myotrauma of diaphragmatic weakness

Conceptually, four distinct types of diaphragm injury as adverse effects of MV are proposed (Fig. 1) [30]:

- Over-assistance myotrauma (disuse atrophy and VIDD) occurs due to excessive respiratory support, coupled with reduced respiratory drive and effort, leading to disuse atrophy and dysfunction. Experimental [31, 32], histological [13, 31], functional [13], and radiological [28] findings provide evidence of over-assistance myotrauma.
- Under-assistance myotrauma (load-induced, concentric contraction) occurs if ventilatory support is insufficient to decrease the load of the diaphragm during periods of increased respiratory demand, as studies in non-critically ill patients and animals suggest [33, 34]. The experimental data lead to the assumption that the resulting high muscle tension during concentric contraction leads to inflammatory infiltration [35] and microscopic disruption of sarcomeres and sarcolemma [28, 33, 34].
- Eccentric myotrauma (load-induced, eccentric contraction) occurs when the diaphragm experiences contractile activity during an expiratory phase with fibre lengthening [30, 36]. This phenomenon emerges during MV dyssynchrony, where the patient's breathing cycles oppose the mechanical ventilator’s work. Experimental data indicate that reverse triggering dyssynchrony [37] has variable effects and current concepts of diaphragm-protective MV do not always rely on avoiding such dyssynchrony [38].
- End-expiratory shortening myotrauma [during high positive end-expiratory pressure (PEEP) levels] manifests as a loss of sarcomere and fibre length, resulting in a reduced expiratory length of the diaphragm. PEEP thus complements cross-sectional atrophy with longitudinal atrophy. Experimental models have confirmed this longitudinal atrophy [39], which can lead to an impaired length–tension ratio of the diaphragm.

In addition to the consequences of MV, diaphragm weakness seems to be accelerated by sepsis, malnutrition, sedation, and disease severity [4, 6]. The impact of neuromuscular relaxants and glucocorticoids is still inconsistent [6, 17, 40, 41]. Although contra-intuitive to the concept of diaphragm unloading resulting in atrophy, the short application of cisatracurium (48 h) in patients with early severe ARDS was associated with a reduced hazard ratio of death at 90 days, while skeletal muscle strength was not influenced [6, 17, 40–42]. However, the use of rocuronium was associated with increased development of contractile dysfunction multiple times in animal studies [24]. Similarly, conflicting data exist on the use of glucocorticoids; results of steroid-induced muscle weakness in ICU patients [6] are contrasted with results of protective effects in animal studies, supposedly by inhibition of the protease calpain [24]. The direct negative mechanistic effect of both drug classes might be counteracted by shortening organ dysfunction (e.g., MV) and, therefore, quicker weaning or recovery.

## PNS for preventive and therapeutical purposes

Neurostimulation of the diaphragm, recently reviewed by Etienne et al., has a long scientific background [43]. Historically, electrical activation of the phrenic nerve inducing diaphragmatic contractions dates back to pivotal studies, with Christoph Hufeland’s 1873 human application during asphyxia in new-borns being the earliest. Further advancements included prolonged stimulation explored by Sarnoff, Hardenbergh, and Whittenberger in 1948 and continued by William Glenn from 1965 onwards [44, 45]. In the following years, surgically implantable devices capable of controlled electrical stimulation of the phrenic nerve emerged as a promising intervention to wean spinal cord injured patients with chronic respiratory insufficiency [46–50].

Concerning a potential therapeutical application of phrenic nerve stimulation, research efforts currently focus on the identification of preventive and therapeutic effects of different stimulation techniques in critically ill patients. Preventive could be the early use of diaphragmatic stimulation to avoid muscle atrophy in the early phase of critical illness and MV, similar to early mobilisation in the skeletal muscle. Early data indicate that temporary PNS is able to ensure continuous diaphragmatic activity during MV [51]. The idea is that by avoiding the disuse of the diaphragm, its mass and function will be preserved, thereby reducing atrophy and dysfunction. Limiting diaphragmatic unloading might result in earlier liberation from MV and, therefore, reduce associated adverse events. An important effect of maintaining diaphragm contraction can be the preservation of aerated lung volume that is normally lost after deep sedation. This effect may improve gas exchange or reduce the risk for the lungs being exposed to risks of VILI.

A therapeutic concept would be diaphragmatic (muscle) training to shorten weaning duration in patients with diaphragmatic weakness. Furthermore, diaphragmatic activation in response to PNS may potentially be used for electrophrenic respiration, i.e. a potential negative pressure ventilation strategy that allows for more physiological ventilation, thereby possibly attenuating the adverse consequences of positive pressure ventilation: PNS-induced ventilation (electrophrenic respiration) may prevent the development of VILI by (1) simply shortening the duration of MV or (2) improving the ratio of desired effects and undesired effects of MV by avoiding its maximal exploitation. A therapeutic concept in critically ill patients would be the application of PNS adjusted to the electrical activity of the patient’s diaphragm, synchronised with spontaneous breathing efforts and supported by non-invasive ventilation, High-Flow or pressure support ventilation.

## Classification of stimulation techniques

**Fig. 2:**
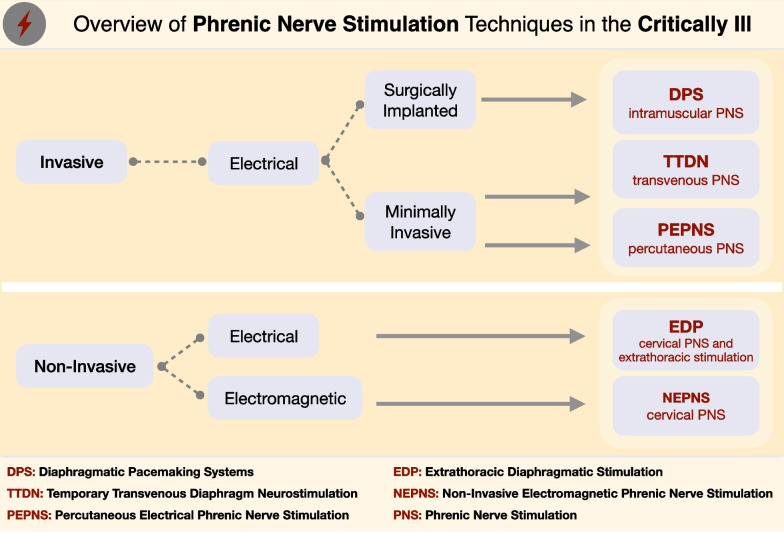
Overview of different phrenic nerve stimulation techniques that have been applied in critically ill patients. The nomenclature of the stimulation techniques was adopted from the studies in which they were first used for this particular study population. Nevertheless, the terms "EDP" [57] and "DPS" [48] have been marginally revised to improve categorisation, differentiation and comprehensibility with respect to the other existing techniques [11, 52–56]

The techniques for PNS can be classified as invasive electrical [48, 52–56], non-invasive electrical [57] or non-invasive (electro)magnetic [11] (Fig. 2). Of note, this review focuses only on PNS, not considering other diaphragm electric stimulation methods that directly stimulate the muscle without significant phrenic nerve transmission (e.g., transcutaneous electrical diaphragmatic stimulation) [49, 58].

### Invasive PNS

Invasive techniques include percutaneous electrical PNS [54], which involves the insertion of a needle or stimulation device close to the phrenic nerves at the neck level. In addition, in critically ill spinal cord injured patients, intramuscular stimulation of the entry points of the phrenic nerves through surgical access has been successfully performed [47]. In the effort to reduce complexity and improve the accessibility of surgically implanted systems for short-term PNS in critically ill patients, recent approaches to simplify surgical implantation have been made. Avoiding nerve dissection, the insertion of removable peri-phrenic electrodes on the terminal segment of the phrenic nerve in between the pericardium and diaphragm was feasible in cadavers. At present, the use of this technique for efficient PNS-induced ventilation has not exceeded proof-of-concept status in animal models [59, 60]. Furthermore, temporary transvenous phrenic nerve stimulation (TTDN) presents a minimally invasive approach wherein both phrenic nerves are stimulated through one central venous line equipped with several electrodes, and the system automatically selects the most suitable combination of electrodes [52].

### Non-invasive PNS

Non-invasive electromagnetic phrenic nerve stimulation (NEPNS), with butterfly-shaped magnetic stimulation coils designed for transcranial application, was performed by Sanders et al. in 2010 [61]. Stimulation was feasible in awake volunteers undergoing continuous positive airway pressure (CPAP) with an external mask; however, the coils were too cumbersome to achieve usability in the clinical setting. Since then, significant progress has been achieved in the implementation of NEPNS. Novel magnetic coils have been designed, specifically adjusted to enhance usability and facilitate diaphragm training for extended ventilation purposes [11, 62] while lacking interference with other ICU devices [63].

Furthermore, Bao et al. innovatively merged non-invasive direct diaphragm electrical stimulation with non-invasive electrical PNS, creating a hybrid approach for non-invasive external diaphragm pacing [57].

### Comparison of electrical and magnetic stimulation techniques

For diagnostic purposes (trans-diaphragmatic pressure assessment), magnetic application surpasses electrical stimulation [64], yet each approach has distinct applications. Magnetic stimulation simplifies phrenic nerve localisation, though it may inadvertently stimulate other structures (e.g., brachial plexus). Electric needle stimulation offers greater precision and selectivity for the target nerve. In contrast, magnetic stimulation might involve the co-activation of auxiliary inspiratory muscles and, additionally, further accessory and ectopic phrenic nerves, generating differing trans-diaphragmatic pressure standard values (20–25% higher with magnetic stimulation due to more negative oesophageal pressure) [65]. Notably, electrical stimulation remains the sole option for patients with pacemakers or implanted medical devices due to interference [65] and is at the present moment the only technique that successfully uses PNS for diaphragm activation in critically ill patients [52, 54, 57, 66].

## Effect of PNS

### Evidence of effects of invasive PNS techniques

Direct phrenic nerve stimulation by surgical access was performed in hemidiaphragms during MV for experimental purposes to demonstrate positive outcomes of diaphragm activation at the molecular level. Positive mitochondrial [55] and oxidative stress effects [56] assessed with high-resolution respirometry and western blotting were reported, suggesting that diaphragm activation during MV could reduce the oxidative stress and counteract autophagy caused by diaphragm paralysis (ameliorating atrophy). Onders et al. explored a diaphragm pacing system to prompt ventilator liberation in 13 critically ill patients with spinal cord injury [47]. Nine (69%) patients were successfully weaned, and in four, tracheostomy was averted, solely relying on PNS. Notably, five patients had the system removed upon full recovery from artificial respiration during follow-up, while three used it continuously for 24-h ventilation post-discharge.

O'Rourke et al. assessed the impact of percutaneous electrical PNS on the work of breathing among patients [54]. The percutaneous stimulation method involved the utilisation of ultrasound to precisely target both phrenic nerves. They demonstrated that in 96.8% (95% CI 96.6–97.0%) work of breathing levels was maintained between 0.2 J/L and 2.0 J/L. This study highlights the feasibility of synchronising PNS with ventilator-induced inspiration while the interaction between the stimulated diaphragm contractions and MV remained harmonic. Percutaneous electrical PNS was associated with a 15% increase in diaphragm thickness after 48 h of application, therefore potentially counteracting diaphragm weakness. However, these results should be interpreted with caution, as diaphragm thickness represents a one-dimensional parameter, which does not necessarily reflect muscle functionality [64].

TTDN was employed by Dres et al. using a multielectrode stimulating central venous catheter for 2–3 training sessions per day in difficult-to-wean patients. While no significant difference in MV time and incidence of death was achieved, significant differences in maximal inspiratory pressure (MIP) were demonstrated between intervention and control during the observation time (+ 16.6 cmH_2_O and + 4.8 cmH_2_O, respectively; *p* = 0.001). The significance was maintained also adjusting for body mass index (BMI) and baseline MIP differences: at day 15 significantly different MIP values were reported with + 11.9 cmH_2_O and + 4.5 cmH_2_O, for intervention and control, respectively (*p* = 0.024) [52].

Comparable changes in MIP were observed in a feasibility trial: among successfully weaned patients, a mean MIP improvement of 19.7 ± 17.9 cmH_2_O (increased by 105%, *p* = 0.02) was reported after TTDN; in the same group, an enhancement of − 63.5 ± 64.4 in the rapid shallow breathing index (RSBI) was documented (*p* = 0.04) [66]. As suggested by the higher muscle strength in the intervention group, these results indicate the potential of PNS-induced diaphragm training to inhibit the progression of diaphragmatic weakness during MV, and consequently enhance the diaphragm force-generating capacity, i.e. diaphragm function.

In a preliminary analysis of a phase one study applying TTDN in mechanically ventilated patients, PNS was innovatively triggered by the ventilator instead of manually and successfully activated the diaphragm in a median of 100% (range 73–100%) of patients [51]. Stimulations were adjusted to achieve a diaphragm electrical activity comparable to low-normal inspiratory effort with an expiratory occlusion pressure of − 5 to − 10 cmH2O. During ventilator-initiated breaths of volume-controlled ventilation, PNS was delivered within 200 ms of the onset and terminated before the end of inspiration.

Further evidence is available from an animal study in sedated pigs receiving invasive electrical PNS with subsequent evaluation of diaphragm muscle thickness and fibre changes [67]. Following intubation and sedation, two interventional groups received TTDN with MV for either every or every other breath, while the control group received MV only. For ventilation, volume-control mode and lung-protective ventilation parameters were used. Stimulations were performed synchronously with ventilator-induced inspiration via a multielectrode stimulation catheter inserted using a subclavian vein catheter. The diaphragm was histologically examined for muscle atrophy, visualising the different fibre types via immunocytochemistry. The combination of PNS and MV resulted in reduced muscle fibre atrophy. In both stimulated groups, muscle fibre cross-sectional area was greater than in the MV-only group. The overall fibre composition of the diaphragm remained unchanged, so no loss of function was found due to different proportions of type 1, 2A and 2X fibres.

### Effect of non-invasive phrenic nerve stimulation techniques

A hybrid method has recently been examined in a pivotal study by Bao et al. in ventilated patients, which included neuromuscular electrical stimulation through an external thoracic belt equipped with electrodes to stimulate the diaphragm, with additional electrical PNS applied through skin electrodes in the neck [57]. This strategy, termed external diaphragmatic pacing, demonstrated increased ultrasound diaphragm excursion (*p* < 0.001) and thickening fraction (*p* = 0.022), indicating successful diaphragm contractions. Clinically, external diaphragmatic pacing seemed to have advantages for liberation from MV.

Regarding single PNS alone, the pioneering study by Sander et al. investigated the feasibility of NEPNS with butterfly-shaped coils in awake and healthy subjects undergoing CPAP with external masks [61]. One limitation of this study on non-narcotised volunteers was the potential presence of a patient's own volitional component in their breath, which could not be excluded. Additionally, the use of cumbersome coils raises problems for critical care applications. Nonetheless, these initial findings provided a basis for exploring magnetic stimulation techniques that target the phrenic nerves.

**Fig. 3:**
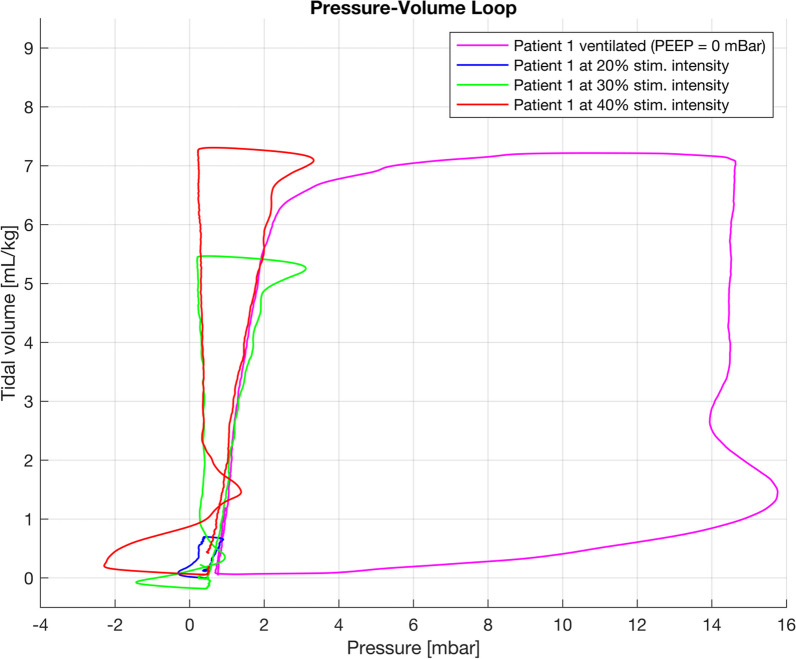
Non-invasive electromagnetic phrenic nerve stimulation in pulmonary healthy patients with absent respiratory drive induces tidal volume with low airway pressures (from the STIMIT I study [11])

Newly engineered compact coils have been developed, tailored specifically for long-term PNS in critical care settings for patients requiring prolonged ventilation support with diaphragm training. Panelli et al. [11] conducted the first feasibility study employing the novel compact coils in intubated and anaesthetised lung-healthy patients scheduled for elective surgery. The phrenic nerves were stimulated bilaterally at the anterior neck level using two stimulation coils connected to a stimulator with a maximum output of 160 Joules (100% stimulation intensity) and a pulse length of 160 µs. The output was technically adjustable from 0–100% in increments of 0.5%. However, due to safety reasons, the intensity was limited to 50%, which resulted in a magnetic flux density of 0.55 Tesla per stimulation coil. Trains of stimulations with frequencies of 25 Hz and durations of 2 s were applied. The study in surgical patients proved that the stimulation coils can efficiently induce phrenic nerve excitation and subsequently activate the diaphragm, independently of voluntary breathing efforts. The mean time to find an adequate stimulation point after ultrasonographic identification of the phrenic nerves was 89 (range 15–441) s. NEPNS achieved a median tidal volume of 7.43 ± 3.06 ml/kg ideal body weight. Furthermore, pressure–volume curves were analysed, revealing an expected negative pressure during inspiration (minimum − 2.7 ± 1.1 cmH_2_O at 40% intensity), corresponding to diaphragm contraction following phrenic stimulation, and positive low pressure during expiration (maximum 3.2 ± 1.1 cmH_2_O at 40%), indicating diaphragm relaxation. The ventilator driving pressure values observed during NEPNS were notably lower than those used conventionally during perioperative MV (Fig. 3).

The additional results characterise NEPNS as a rapidly applicable technique: the time required to find a suitable stimulation point was approximately 90 s. The magnetic field generated appears to allow some flexibility in the positioning of the stimulation coil. Even with a 0.5 to 1 cm stimulation coil movement in anterior–posterior direction along the neck, away from the previously identified adequate stimulation point, diaphragm contractions could still be induced. Data regarding the implementation of NEPNS in anaesthetised and morbidly obese patients are currently anticipated (https://clinicaltrials.gov/; NCT05107167).

Another research group, Mueller et al., used the same coil technology to investigate the safety aspects in healthy intubated subjects under anaesthesia. They reported tidal volumes of up to 279 ml (ranging from 80 to 557 ml) with a stimulation intensity of 40% [62]. Notably, both studies recorded no severe adverse events related to the stimulation intervention. Only a few minor adverse events, such as skin redness, were noted and typically resolved within six hours post-intervention.

These findings collectively highlight the potential of this technology for safe, non-invasive, and electromagnetic PNS and show its capability to generate sufficient tidal volumes to ventilate lung-healthy patients without MV support. The absence of severe adverse events and the minimal occurrence of minor events further support exploring this approach for research on preventing diaphragm weakness and respiratory support during MV. Moreover, the finding of low driving pressures during stimulations may be an additional indication of the safety of NEPNS because the pressure values align with the recommended airway pressure limits for lung-protective MV, as per the latest ARDS guidelines [68], but further research and analysis of the evoked lung pressures is necessary. Possibly, NEPNS could be employed as a safe approach to provide respiratory support, ensuring compliance with the established safety thresholds for ventilation during critical care. Additionally, an assessment of the long-term application of NEPNS in a critical care setting is required to obtain crucial data regarding the durability of the method for inducing continuous diaphragm activation. Feasibility data on NEPNS in critically ill patients (https://clinicaltrials.gov/; NCT05238753) have recently been presented in abstract form [69].

### Prevention of VILI

The concept of prevention of VILI using PNS is based on reducing MV duration or its extent of detrimental consequences. PNS-induced diaphragm activation preventing diaphragm weakness might result in lower ventilator dependency with improved liberation and reduced weaning duration. However, the primary expected outcome of PNS on the development of VILI is to prevent or mitigate lung injury by either replacing or supporting MV. This concept is driven by the idea that lower levels of mechanical power are induced with PNS rather than with MV, possibly due to greater similarities with physiological respiration or to the reaeration of the lung due to diaphragm contraction. Driving and transpulmonary pressures are expected to be lower than with MV, even when lung volume is increased. Airway pressures, but not transpulmonary pressures, were recently shown to be lower using NEPNS in lung-healthy anaesthetised patients. Further data are needed to determine if this is true for lung-distending pressures [11]. Consequently, the consistent combination of MV with PNS for every (other) breath might result in a lower risk of VILI development; however, it has not been investigated in critically ill patients.

An experimental study investigated the application of TTDN supplementing MV in a preclinical ARDS model in 24 pigs allocated to four groups ((1) PNS every breath, (2) PNS every other breath, (3) MV without stimulation, and (4) no ventilation and stimulation). After intubation, sedation, and the onset of volume-control ventilation (depending on group allocation), lung injury was induced with oleic acid via a pulmonary artery catheter until an arterial tension of oxygen/fraction of inspired oxygen ratio (PaO_2_/FiO_2_ ratio) of ≤ 200 mmHg was achieved. PNS was applied using TTDN via a subclavian vein catheter synchronised with the ventilator-induced inspiration. Experimental parameters, including transpulmonary pressure and static compliance, end-expiratory lung volume loss, and extravascular lung water, were measured via a respiratory monitor connected to a nasogastric catheter, electrical impedance tomography and pulse contour analysis, respectively. Post-mortem lung tissue biopsies were histologically evaluated to assess the extent of lung jury. Further processing was performed for protein and cytokine analysis [70]. This combined PNS and MV approach displayed benefits over MV regarding lung injury extent. TTDN, together with volume-controlled ventilation, improved pulmonary function, reduced inflammatory processes, and limited lung oedema, resulting in lower lung injury scores, driving pressures and mechanical power.

These preliminary findings need validation for critically ill patients but provide rare necessary evidence supporting PNS as a strategy against VILI [67, 70].

## Future perspectives of phrenic nerve stimulation

Following the aforementioned promising indications of PNS in attenuating diaphragm atrophy among critically ill patients, the potential application of electrophrenic respiration by PNS appears feasible in the ICU context. Some challenges, however, must be addressed. Manual synchronisation of stimulation during spontaneous breathing should be avoided to minimise ventilation cycle mismatches. Incorporating automatic recognition of the breathing cycle [51], potentially using machine learning, can ensure timely stimulation during inspirations. PNS might allow to remain within lower inspiratory pressure settings of ventilatory support devices, while continuously ensuring adequate tidal volumes [11]. Thereby the technique could be proposed as a support form that might offer respiratory assistance, potentially inducing a lower risk of excessive driving pressure and transpulmonary pressure, as compared with spontaneous breathing efforts using ventilatory support modes [71, 72] or MV itself, therefore mitigating VILI. The stimulation dosage required for this concept remains unclear, and it is crucial to avoid overloading a diaphragm conditioned by critical illness. In essence, the dosage should stay in the physiological range of respiratory work and be adjusted to the individual's respiratory effort and the respiratory demands resulting from reduced MV.

## Conclusions

The administration of PNS has started to demonstrate feasibility and safety in the critical care setting. Among various existing, non-standardised stimulation techniques, electrically invasive techniques indicate beneficial effects on respiratory strength. PNS-driven diaphragm contractions can generate adequate ventilation in intubated patients, whose cyclical changes in lung volumes are characterised by low transpulmonary pressures with a low risk of lung stress. This protective effect of PNS on the lung may be particularly beneficial in the context of critical care ventilation, where minimising VILI is a primary concern. However, existing data are still rare and primarily found in preclinical studies. A combination of MV and TTDN in an animal model was able to limit the continuous lung stress and strain of MV alone.

PNS for diaphragmatic weakness and VILI prevention is a field of active research; however, as of the present moment, only pilot results have been reported. NEPNS notably shows low airway pressure ranges possibly compatible with a low risk of excessive transpulmonary pressure and associated lung injury. However, to solidify these findings, studies with a high level of evidence are necessary to validate the potential benefits of PNS in critically ill patients. Currently, phrenic nerve stimulation for diaphragm atrophy, VIDD and VILI therapy remains a highly active research field.

## Data Availability

Not applicable.

## Abbreviations

ARDS: Acute respiratory distress syndrome
CPAP: Continuous positive airway pressure
ICU: Intensive care unit
ICUAW: Intensive care unit acquired weakness
MIP: Maximal inspiratory pressure
MV: Mechanical ventilation
NIV: Non-invasive ventilation
NEPNS: Non-invasive electromagnetic phrenic nerve stimulation
PEEP: Positive end-expiratory pressure
TTDN: Temporary transvenous diaphragm neurostimulation
VIDD: Ventilator-induced diaphragm dysfunction
VILI: Ventilator-induced lung injury
